# The Impact of Breast Cancer Treatment Delays on Survival Among South African Women

**DOI:** 10.1093/oncolo/oyab054

**Published:** 2022-01-31

**Authors:** Yoanna S Pumpalova, Oluwatosin A Ayeni, Wenlong Carl Chen, Ines Buccimazza, Sharon Cačala, Laura W Stopforth, Hayley A Farrow, Witness Mapanga, Sarah Nietz, Boitumelo Phakathi, Maureen Joffe, Valerie McCormack, Judith S Jacobson, Katherine D Crew, Alfred I Neugut, Paul Ruff, Herbert Cubasch, Daniel S O’Neil

**Affiliations:** Department of Medicine, Vagelos College of Physicians and Surgeons, Columbia University, New York, NY, USA; Noncommunicable Diseases Research Division, Wits Health Consortium (PTY) Ltd, Johannesburg, South Africa; SAMRC/Wits Developmental Pathways to Health Research Unit, Department of Paediatrics, Faculty of Health Sciences, University of Witwatersrand, Johannesburg, South Africa; South Africa Medical Research Council Common Epithelial Cancers Research Centre, Faculty of Health Sciences, University of Witwatersrand, Johannesburg, South Africa; Noncommunicable Diseases Research Division, Wits Health Consortium (PTY) Ltd, Johannesburg, South Africa; National Cancer Registry, National Health Laboratory Service, Johannesburg, South Africa; Sydney Brenner Institute for Molecular Bioscience, Faculty of Health Sciences, University of the Witwatersrand, Johannesburg, South Africa; Noncommunicable Diseases Research Division, Wits Health Consortium (PTY) Ltd, Johannesburg, South Africa; Department of Surgery, Faculty of Health Sciences, University of the Witwatersrand, Johannesburg, South Africa; Noncommunicable Diseases Research Division, Wits Health Consortium (PTY) Ltd, Johannesburg, South Africa; Department of Specialized Surgery, Inkosi Albert Luthuli Central Hospital, Durban and Ngwelezane Hospital, Empangeni, University of KwaZulu-Natal, Empangeni, KwaZulu-Natal, South Africa; Noncommunicable Diseases Research Division, Wits Health Consortium (PTY) Ltd, Johannesburg, South Africa; Departments of Surgery and Radiation Oncology, Grey’s Hospital, University of KwaZulu-Natal, Pietermaritzburg, KwaZulu-Natal, South Africa; Departments of Surgery and Radiation Oncology, Grey’s Hospital, University of KwaZulu-Natal, Pietermaritzburg, KwaZulu-Natal, South Africa; Noncommunicable Diseases Research Division, Wits Health Consortium (PTY) Ltd, Johannesburg, South Africa; Division of Medical Oncology, Department of Medicine, Faculty of Health Sciences, University of the Witwatersrand, Johannesburg, South Africa; National Cancer Registry, National Health Laboratory Service, Johannesburg, South Africa; Department of Surgery, Faculty of Health Sciences, University of the Witwatersrand, Johannesburg, South Africa; Noncommunicable Diseases Research Division, Wits Health Consortium (PTY) Ltd, Johannesburg, South Africa; Department of Surgery, Faculty of Health Sciences, University of the Witwatersrand, Johannesburg, South Africa; Noncommunicable Diseases Research Division, Wits Health Consortium (PTY) Ltd, Johannesburg, South Africa; SAMRC/Wits Developmental Pathways to Health Research Unit, Department of Paediatrics, Faculty of Health Sciences, University of Witwatersrand, Johannesburg, South Africa; South Africa Medical Research Council Common Epithelial Cancers Research Centre, Faculty of Health Sciences, University of Witwatersrand, Johannesburg, South Africa; Environment and Lifestyle Epidemiology Branch, International Agency for Research on Cancer (IARC/WHO), Lyon, France; Herbert Irving Comprehensive Cancer Center, Vagelos College of Physicians and Surgeons, Columbia University, New York, NY, USA; Department of Epidemiology, Mailman School of Public Health, Columbia University, New York, NY, USA; Department of Medicine, Vagelos College of Physicians and Surgeons, Columbia University, New York, NY, USA; Herbert Irving Comprehensive Cancer Center, Vagelos College of Physicians and Surgeons, Columbia University, New York, NY, USA; Department of Medicine, Vagelos College of Physicians and Surgeons, Columbia University, New York, NY, USA; Herbert Irving Comprehensive Cancer Center, Vagelos College of Physicians and Surgeons, Columbia University, New York, NY, USA; Department of Epidemiology, Mailman School of Public Health, Columbia University, New York, NY, USA; Noncommunicable Diseases Research Division, Wits Health Consortium (PTY) Ltd, Johannesburg, South Africa; SAMRC/Wits Developmental Pathways to Health Research Unit, Department of Paediatrics, Faculty of Health Sciences, University of Witwatersrand, Johannesburg, South Africa; Division of Medical Oncology, Department of Medicine, Faculty of Health Sciences, University of the Witwatersrand, Johannesburg, South Africa; Noncommunicable Diseases Research Division, Wits Health Consortium (PTY) Ltd, Johannesburg, South Africa; South Africa Medical Research Council Common Epithelial Cancers Research Centre, Faculty of Health Sciences, University of Witwatersrand, Johannesburg, South Africa; Department of Surgery, Faculty of Health Sciences, University of the Witwatersrand, Johannesburg, South Africa; Sylvester Comprehensive Cancer Center and Department of Medicine, Miller School of Medicine, University of Miami, Miami, FL, USA

**Keywords:** global oncology, breast cancer, treatment delays, South Africa

## Abstract

**Background:**

In high-income settings, delays from breast cancer (BC) diagnosis to initial treatment worsen overall survival (OS). We examined how time to BC treatment initiation (TTI) impacts OS in South Africa (SA).

**Methods:**

We evaluated women enrolled in the South African BC and HIV Outcomes study between July 1, 2015 and June 30, 2019, selecting women with stages I-III BC who received surgery and chemotherapy. We constructed a linear regression model estimating the impact of sociodemographic and clinical factors on TTI and separate multivariable Cox proportional hazard models by first treatment (surgery and neoadjuvant chemotherapy (NAC)) assessing the effect of TTI (in 30-day increments) on OS.

**Results:**

Of 1260 women, 45.6% had upfront surgery, 54.4% had NAC, and 19.5% initiated treatment >90 days after BC diagnosis. Compared to the surgery group, more women in the NAC group had stage III BC (34.8% vs 81.5%). Living further away from a hospital and having hormone receptor positive (vs negative) BC was associated with longer TTI (8 additional days per 100 km, *P = .*003 and 8 additional days, *P = .*01, respectively), while Ki67 proliferation index >20 and upfront surgery (vs NAC) was associated with shorter TTI (12 and 9 days earlier; *P = .*0001 and.007, respectively). Treatment initiation also differed among treating hospitals (*P < .*0001). Additional 30-day treatment delays were associated with worse survival in the surgery group (HR 1.11 [95%CI 1.003-1.22]), but not in the NAC group.

**Conclusions:**

Delays in BC treatment initiation are common in SA public hospitals and are associated with worse survival among women treated with upfront surgery.

## Teaser

This article examines how time to breast cancer treatment initiation affects overall survival in South African public hospitals.

**Implications for Practice**

We show that among women with non-metastatic breast cancer enrolled in the South African Breast Cancer and HIV Outcomes Study, delays in breast cancer treatment initiation are generally associated with worse survival. Our work forms an evidence base in support of the World Health Organization Global Breast Cancer Initiative, which aims to improve breast cancer survival, and has identified timely treatment as one of 3 key mechanisms necessary to achieve this goal.

## Introduction

Using population-based registries, the 2018 CONCORD-3 trial estimated the 5-year breast cancer (BC) survival in South Africa (SA) to be 40.1%, compared with 90.2% in the US.^1^ More recently, the African Breast Cancer-Disparities in Outcomes (ABC-DO) prospective cohort study, conducted in Namibia, Nigeria, South Africa, Uganda and Zambia and published in 2020, estimated the 3-year overall survival (OS) for BC (all stages) to be 50% (95% confidence interval (CI) 48-53), with large variations by race and country (90% survival in white Namibian women compared with 56% in Black Namibian women and 63% in Black South African women).^2^

Poor BC survival in sub-Saharan Africa (SSA) has been attributed to advanced stage at diagnosis, aggressive tumor biology, high rates of HIV, and difficult to access or suboptimal treatment.^2-5^ Several studies from the region have identified socioeconomic, cultural, and health systems barriers that correlate with delays in BC diagnosis and BC treatment initiation, but have not reported on the impact of treatment delays on survival in SSA.^3,6-14^

Prior studies conducted outside SSA suggest that increased time to treatment initiation (TTI), defined as time from BC diagnosis to first treatment, adversely impacts survival.^15-19^ In a retrospective cohort of 2045 nonmetastatic Korean BC patients, surgical delay of >12 weeks versus ≤4 weeks after diagnosis was associated with worse OS (hazard ratio, HR 1.91, 95%CI 1.06-3.49).^18^ Large studies from the US using the National Cancer Data Base (NCDB) and the Surveillance, Epidemiology, and End Results (SEER)-Medicare database have shown similar results, although the observed effect of TTI on OS was smaller in magnitude (HR 1.14 [95%CI 1.09-1.20] for risk of death from any cause with delay of >12 weeks from diagnosis to surgery versus ≤12 weeks^17^ and HR of 1.09 [95%CI 1.06-1.13] for increase in death from any cause for each additional 30-day delay between diagnosis and surgery).^16^ In a US retrospective cohort, delays of >60 days from diagnosis to start of neoadjuvant chemotherapy (NAC) were also associated with greater risk of death from any cause (HR of 1.28 [95%CI 1.06-1.54] compared with initiating NAC within 30 days of diagnosis).^19^

In March 2021, recognizing the global rise in BC incidence and mortality, the World Health Organization (WHO) launched the Global Breast Cancer Initiative, which aims to reduce global BC mortality by supporting governments in 3 areas: (1) promoting BC awareness, (2) improving timely BC diagnosis and treatment, and (3) providing comprehensive BC treatment and supportive care.^20^ Survival gap apportionment analysis of the ABC-DO cohort concluded that, in SSA, population downstaging of BC and improving access to therapy (defined as a patient undergoing both surgery and chemotherapy within 12 months of diagnosis) would have the greatest impact on improving OS, averting an estimated one third of observed deaths.^2^ In this study, we investigated the association of TTI, defined as time from BC diagnosis to first treatment (surgery or NAC), with OS among women with localized BC enrolled in the South Africa Breast Cancer and HIV Outcomes (SABCHO) study. Although such an association is observed in high-income countries, it is not known whether TTI is associated with survival in SSA, where late-stage disease and variable quality of cancer care are major drivers of poor BC outcomes.

## Methods

### Data Source

The SABCHO study is a prospective cohort study that began enrolling women from 6 public hospitals in SA in February 2015; a variety of demographic, socioeconomic, and clinical data have been prospectively collected for over 3500 women with BC, as previously described.^21^ Women eligible for the SABCHO study were >18 years of age, had newly diagnosed BC, had no history of other cancers, received BC treatment at a study hospital and signed informed consent. The participating hospitals were Chris Hani Baragwanath Academic Hospital in Johannesburg (CHBAH); Charlotte Maxeke Johannesburg Academic Hospital in Johannesburg (CMJAH); Grey’s Hospital in Pietermaritzburg (GH); Ngwelezana Hospital in Empangeni (NH); and Addington Hospital/Inkosi Albert Luthuli Central Hospital in Durban (analyzed as one site, ALH). The African Breast Cancer Disparities in Outcomes study (ABC-DO) recruited patients from CHBAH at the same time as the SABCHO study; thus, many of the patients recruited from this site participated in both studies.^22^

### Patient Population

For this analysis, we identified a sub-cohort of women from the SABCHO cohort; we included participants who enrolled between June 30, 2015 and July 1, 2019, and were diagnosed with American Joint Committee on Cancer, 7th edition, stage I, II, or III BC. Prior work from our group revealed that 62.3% of women with localized and locally advanced BC in the SABCHO cohort received NAC as their first treatment;^23^ thus in the current study we included women who received either NAC or surgery as first treatment for localized BC, followed by surgery or adjuvant chemotherapy, respectively. Women who did not initiate adjuvant chemotherapy after surgery (due to default, or because it was not clinically indicated), women who did not undergo surgery after NAC, and women who failed to initiate radiation therapy after breast conserving surgery were excluded to achieve a comparably treated group that allowed for survival analysis of all included women. All women had complete blood count, liver function tests, basic metabolic panel, chest X-ray, and abdominal ultrasound as part of routine staging work up. Bone scans, other X-rays, and CT scans were added as indicated by patient symptoms or earlier studies.

### Covariates and Outcomes

Data were collected on date of BC diagnosis (date of first diagnostic biopsy), place of BC diagnosis (SABCHO study site vs. other), age at diagnosis, self-reported ethnicity, home address, marital status, highest level of education, self-reported time between first breast symptoms and BC diagnosis, BC clinical stage, tumor grade, estrogen and progesterone receptor (ER/PR) status, human epidermal growth factor receptor 2 (HER2) expression, Ki67 proliferation index, HIV status, BC treatment hospital, first BC treatment received (surgery or NAC), date of BC surgery, and date of first cycle of NAC. The longitudes and latitudes of patients’ addresses were ascertained via iTouchMap.com, and the distance from their home to the treating hospital was calculated using the Vincenty formula, which calculates the straight-line distance over a spherical surface.^24-26^ Our team previously developed and validated a “Barriers to Breast Cancer Care” questionnaire, which was completed by all participants at time of enrollment into SABCHO; a BC knowledge score (KS) was assigned based on participants’ responses to the 7 BC Knowledge questions included in the questionnaire.^13^ Participants rated the following 7 BC statements on a 5-point scale (‘Strongly Disagree’ to ‘Strongly Agree’): (1) a person can catch BC from someone else, (2) cancer can run in families and be inherited, (3) BC can be caused by an injury to the breast, (4) BC can be caused by a curse, (5) a painless lump can be a sign of BC, (6) a painful lump can be a sign of BC, (7) fluid coming from the nipple can be a sign of BC. For statements 2, 5, 6, and 7, patients received 1 or 2 points when they agreed or strongly agreed, respectively, and lost 1 or 2 points when they disagreed or strongly disagreed, respectively. Vice versa scoring was applied for statements 1, 3, and 4. Breast cancer KS was the sum of all points (range: −14 to 14).

Treatment initiation was defined as the number of days elapsed from the date of BC diagnosis to the date of first BC treatment (upfront surgery or day 1 of NAC). Patients were assigned to delay groups by TTI: ≤90 and >90 days. Outcome data were collected through June 30, 2020. Patients who did not routinely attend clinic were contacted every 3 months after enrollment to determine vital status. If the patient, next of kin, and other provided person of contact were unable to be reached for 2 consecutive follow-up calls, we searched publicly available administrative data to determine the patient’s vital status. If no additional information about vital status could be obtained, the patient was censored at last date she was known to be alive. Overall survival was defined as time from first BC treatment (date of surgery or first day of NAC) to death from any cause.

### Statistical Analysis

We stratified the cohort by first treatment type (surgery and NAC) and used the Kruskal-Wallis test (nominal variables) and the Spearman test (continuous and ordinal variables) to compare frequency distributions of the above-listed demographic and clinical variables between patients by TTI (≤90 and >90 days). We built a multiple linear regression model to estimate the impact of those same variables on TTI in the entire cohort. That model included all the variables with a *P*-value <.05 on crude analysis; HIV status was included a priori based on the SABCHO study’s overarching aim to understand the impact of comorbid HIV on BC. For the multiple linear regression model, TTI was expressed as a continuous variable in discrete days.

We constructed separate -Kaplan-Meier curves for upfront surgery and NAC to estimate OS, comparing patients by TTI delay groups (≤90 and >90 days). Overall survival was calculated on a time from treatment start scale, as defined above. Assuming unequal sample sizes (allocation ratio 5:1) and a total sample size of 500, we had 80% power to detect a crude HR of 1.4 for TTI >90 days versus ≤90 days for each first treatment type group (surgery and NAC), with type 1 error set at 5%.

Using Cox proportional hazards models, we estimated the HR for the effect of TTI on OS. For the Cox proportional hazards models, TTI was expressed as a continuous variable in 30-day increments and OS was calculated on a time from treatment start scale, as defined above. Separate models were constructed for each first treatment type group and adjusted for patient factors (age at diagnosis, ethnicity (black vs other), distance from study hospital, time from first breast symptoms to BC diagnosis (<90 vs ≥90 days)), health system factors (treating hospital (CHBAH vs others) and diagnosis location (SABCHO site vs other)) and clinical factors (BC stage (stages I-II vs stage III), grade (1-2 vs. 3), ER/PR status, HER2 receptor status, Ki67 proliferation index (≤20% vs >20%), and HIV status at diagnosis).

We then performed 2 exploratory survival analyses. Examination of the -Kaplan-Meier curves revealed many mortality events in the first year after treatment initiation for localized BC in our cohort; we suspect that this high early mortality rate was due to under-staging, whereby women with metastatic BC may have been misclassified as having localized BC because computerized tomography and magnetic resonance imaging were not routinely used in study hospitals. To reduce the risk of such misclassification bias we conducted a 1-year conditional survival analysis, excluding women who died or were lost to follow up within 1 year of their BC diagnosis (Table 4, exploratory analysis 1). Second we noted that more than 50% of women treated at Addington and/or Inkosi Albert Luthuli Central Hospitals (ALH) experienced treatment delays >90 days, which may have limited our model’s capacity to control for the impact of hospital factors on survival. To reduce this effect, we repeated the survival analysis, excluding women treated at ALH (Table 4, exploratory analysis 2).

All statistical analyses were done using SAS version 9.4. The study was approved by the University of the Witwatersrand Human Research Ethics Committee and the Institutional Review Board of Columbia University.

## Results

### Baseline Characteristics

From July 1, 2015 through June 30, 2019, 3081 women enrolled in the SABCHO cohort. Of those women, we sequentially excluded the following: 23 patients with missing HIV status, 612 diagnosed with stage IV BC, 49 with bilateral BC, 12 with encapsulated or sarcomatoid histology, 4 with missing stage, 581 who did not undergo curative-intent BC surgery, 364 who did not initiate curative-intent chemotherapy, 106 who did not receive radiation after breast conserving surgery, and 109 who initiated endocrine therapy or radiation therapy as first BC treatment. In total, 1260 women were included in our analysis.

**Figure 1.**
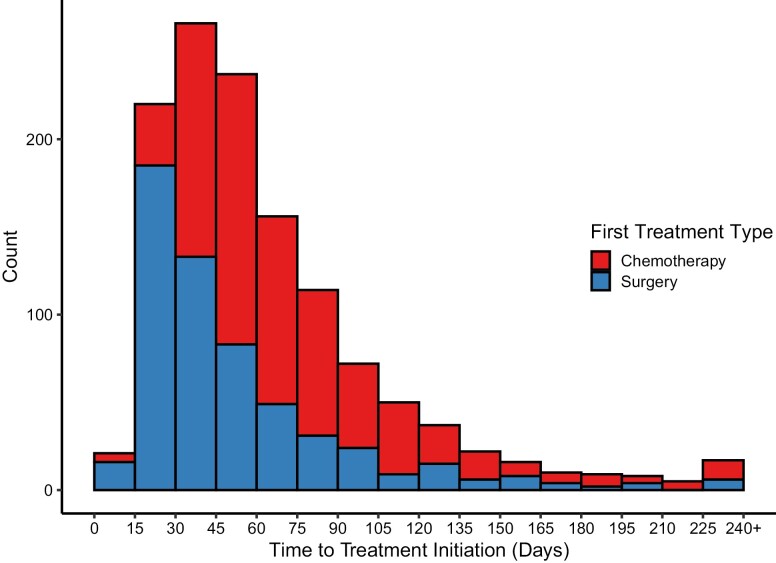
Frequency distribution by time from diagnosis to first treatment initiation (surgery or neoadjuvant chemotherapy) for women in the SABCHO cohort with stages I-III breast cancer.

Baseline characteristics of the women included in our analysis are presented in Table 1. Most women in our cohort were treated with NAC (54.4%). In the upfront surgery group, 86.4% of women had surgery within 90 days of BC diagnosis, compared with 75.5% in the NAC group. In the surgery first group, the median TTI was 40 days after BC diagnosis (interquartile range (IQR) 27-63), with a median of 35 days (IQR 26-51) and 127 days (IQR 102-202) for the ≤90 and >90-day delay groups, respectively. In the NAC group, the median TTI was 63 days (IQR 45-90), with a median of 53 (IQR 41-68) and 119 days (IQR 104-149), for the ≤90 and >90-day delay groups, respectively. A histogram depicting frequency distribution of women by TTI for the 2 first treatment type groups is available in Fig. 1.

**Table 1.** Socio-demographic and clinical characteristics of women in the SABCHO cohort treated for stages I-III breast cancer, by first treatment and time to treatment initiation.

First treatment	Surgery						Chemotherapy					
Delay group	≤90 days		>90 days		Total	*P*-value	≤90 days		>90 days		Total	*P*-value
	*N* = 497		*N* = 78		*N* = 575		*N* = 517		*N* = 168		*N* = 685	
**Age (years)**												
<30	6	1.2%	1	1.3%	7	0.15^a^	15	2.9%	6	3.6%	21	0.51^a^
30-40	51	10.3%	8	10.3%	59		82	15.9%	29	17.3%	111	
40-50	134	27.0%	15	19.2%	149		155	30.0%	51	30.4%	206	
50-60	128	25.8%	19	24.4%	147		137	26.5%	43	25.6%	180	
≥60	178	35.8%	35	44.9%	213		128	24.8%	39	23.2%	167	
Ethnicity												
Black	417	83.9%	60	76.9%	477	**.001** ^b^	405	78.3%	125	74.4%	530	**.04** ^b^
Asian	28	5.6%	14	17.9%	42		55	10.6%	31	18.5%	86	
White	27	5.4%	2	2.6%	29		34	6.6%	7	4.2%	41	
Colored	25	5.0%	2	2.6%	27		23	4.4%	5	3.0%	28	
Distance from hospital												
<20 km	264	53.1%	27	34.6%	291	**<.0001** ^a^	265	51.3%	74	44.0%	339	**.02** ^**a**^
20-50 km	152	30.6%	15	19.2%	167		175	33.8%	48	28.6%	223	
50-100 km	32	6.4%	13	16.7%	45		29	5.6%	29	17.3%	58	
≥100 km	49	9.9%	23	29.5%	72		48	9.3%	17	10.1%	65	
Education												
None/some primary	55	11.1%	18	23.7%	73	**.003** ^a^	54	10.5%	27	16.2%	81	**.002** ^a^
Primary	218	44.0%	35	46.1%	253		204	39.5%	82	49.1%	286	
Secondary	181	36.5%	18	23.7%	199		213	41.3%	43	25.7%	256	
Postsecondary	42	8.5%	5	6.6%	47		45	8.7%	15	9.0%	60	
Missing	1		2		3		1		1		2	
Time to BC diagnosis												
<90 days	358	72.5%	49	62.8%	407	.08^b^	330	64.0%	104	62.7%	434	.76^b^
≥90 days	136	27.5%	29	37.2%	165		186	36.0%	62	37.3%	248	
Missing	3				3		1		2		3	
Stage												
I	33	6.6%	7	9.0%	40	.53^a^	2	0.4%	0	0.0%	2	**.004** ^a^
II	295	59.4%	40	51.3%	335		81	15.7%	44	26.2%	125	
III	169	34.0%	31	39.7%	200		434	83.9%	124	73.8%	558	
Grade												
1-2	313	63.2%	59	76.6%	372	**.02** ^a^	251	51.4%	96	64.4%	347	**.001** ^a^
3	182	36.8%	18	23.4%	200		237	48.6%	53	35.6%	290	
Missing	2		1		3		29		19		48	
ER/PR												
Positive	384	77.4%	71	91.0%	455	**.006** ^b^	375	72.8%	117	70.1%	492	.49^b^
Negative	112	22.6%	7	9.0%	119		140	27.2%	50	29.9%	190	
Missing	1				1		2		1		3	
HER2												
Negative	374	75.4%	61	78.2%	435	.59^b^	342	66.5%	113	68.1%	455	.71^b^
Positive	122	24.6%	17	21.8%	139		172	33.5%	53	31.9%	225	
Missing	1				1		3		2		5	
Ki67												
≤20%	165	33.2%	41	52.6%	206	**.0009** ^b^	178	34.4%	93	55.4%	271	**<.0001** ^b^
>20%	332	66.8%	37	47.4%	369		339	65.6%	75	44.6%	414	
HIV												
Negative	391	78.7%	59	75.6%	450	.55^b^	401	77.6%	120	71.4%	521	.11^b^
Positive	106	21.3%	19	24.4%	125		116	22.4%	48	28.6%	164	
BC KS^c^												
Mean (SD)	6.9	3.6	6.7	3.6		**.01** ^a^	6.5	3.5	5.7	3.1		**<.0001** ^a^
Diagnosis year												
2015	59	11.9%	6	7.7%	65	.3^a^	49	9.5%	22	13.1%	71	**.04** ^a^
2016	149	30.0%	27	34.6%	176		182	35.2%	65	38.7%	247	
2017	141	28.4%	34	43.6%	175		122	23.6%	43	25.6%	165	
2018	124	24.9%	8	10.3%	132		142	27.5%	32	19.0%	174	
2019	24	4.8%	3	3.8%	27		22	4.3%	6	3.6%	28	
Hospital												
CHBAH	259	52.1%	12	15.4%	271	**<.0001** ^b^	199	38.5%	24	14.3%	223	**<.0001** ^b^
CMJAH	94	18.9%	11	14.1%	105		159	30.8%	19	11.3%	178	
GH	134	27.0%	32	41.0%	166		69	13.3%	13	7.7%	82	
ALH	9	1.8%	21	26.9%	30		84	16.2%	93	55.4%	177	
NH	1	0.2%	2	2.6%	3		6	1.2%	19	11.3%	25	
Place of diagnosis												
SABCHO site	373	78.4%	59	75.6%	432	.59^b^	427	85.7%	129	84.3%	556	.66^b^
Other	103	21.6%	19	24.4%	122		71	14.3%	24	15.7%	95	
Missing	21				21		19		15		34	
Place of surgery												
SABCHO site	379	83.1%	53	86.9%	432	.46^b^	432	96.4%	121	98.4%	553	.27^b^
Other	77	16.9%	8	13.1%	85		16	3.6%	2	1.6%	18	
Missing	41		17		58		69		45		114	

Bold text indicateds statistically significant.

a Spearman test.

b Kruskal-Wallis test.

c Breast cancer knowledge score ranges: −14,14.

BC = breast cancer; KS = knowledge score; SABCHO = South Africa Breast Cancer and HIV Outcomes study; CHBAH = Chris Hani Baragwanath Academic Hospital; CMJAH = Charlotte Maxeke Johannesburg Academic Hospital; GH = Grey’s Hospital; ALH = Addington Hospital/Inkosi Albert Luthuli Central Hospital; NH = Ngwelezana Hospital; ER/PR = estrogen receptor/progesterone receptor.

Most of our cohort self-identified as Black (79.9%), lived within 50 km of a SABCHO site (81.0%), presented with stage III BC (60.2%), and had an ER/PR positive tumor (75.2%). Compared to women who received upfront surgery, women treated with NAC were younger (median age 55.2 vs 50.1 years) and more likely to have stage III BC (34.8% vs 81.5%), ER/PR negative BC (20.7% vs 27.9%) and HER2 positive BC (24.2% vs 33.1%).

### Factors Associated with BC Treatment Delays

On crude analysis, ethnicity, distance from treatment hospital, level of education, BC grade, Ki67 index, BC KS, and treatment hospital were associated with TTI among women treated with upfront surgery or NAC (Table 1). In the upfront surgery group, BC hormone receptor status was also associated with TTI, while in the NAC group, BC stage and year of diagnosis were also associated with TTI (Table 1). All significant variables on crude analysis were included in the linear regression model.

The variables included in the linear regression model explained 19% of the variance in TTI (Table 2). Treatment at ALH, GH, and NH was correlated with longer treatment delays, relative to CHBAH; BC treatment at ALH or NH was associated with the longest delays relative to CHBAH (46 and 44 additional days respectively; both *P < .*0001). Living further away from a treatment hospital and having ER/PR positive BC were also associated with longer treatment delays (8 additional days per 100 additional km, *P = .*003 and 8 additional days for ER/PR positive vs negative BC, *P = .*01). A Ki-67 proliferation score >20% and upfront surgery (vs NAC) were associated with earlier treatment initiation (earlier treatment by 12 and 9 days, *P = .*0001 and .007, respectively). Neither black ethnicity nor HIV infection status was significantly associated with TTI, but both showed a trend toward delayed treatment (7 and 6 days, respectively, both *P = .*08).

**Table 2.** Multiple regression model for risk factors associated with time to treatment initiation among women in the SABCHO cohort with stages I-III breast cancer and treated with chemotherapy and surgery.

Coefficient	Unstandardized β (days)	Std. error (days)	Standardized β	*P*-value
Black Ethnicity	6.64	3.75	0.05	.08
Distance from hospital	**0.08**	0.03	0.09	**.003**
Increasing education	−2.00	1.76	−0.03	.26
Increasing stage	3.02	2.72	0.03	.27
Grade 3	1.66	2.93	0.02	.57
ER/PR positive	**8.29**	3.22	0.07	**.01**
Ki-67 >20%	−**11.53**	2.96	−0.11	**.0001**
Increasing BC KS	−0.49	0.40	−0.03	.22
Study hospital (baseline: CHBAH)				
CMJAH	4.52	3.56	0.04	.2
ALH	**45.50**	4.72	0.32	**<.0001**
GH	**9.38**	4.18	0.08	**.02**
NH	**43.51**	10.15	0.12	**<.0001**
Increasing year of diagnosis	0.65	1.30	0.01	.62
Upfront surgery	−**8.70**	3.24	−0.09	**.007**
HIV positive	5.63	3.25	0.05	.08

Bold text indicateds statistically significant.

SABCHO = South Africa Breast Cancer and HIV Outcomes study; CHBAH = Chris Hani Baragwanath Academic Hospital; CMJAH = Charlotte Maxeke Johannesburg Academic Hospital; GH = Grey’s Hospital; ALH = Addington Hospital/Inkosi Albert Luthuli Central Hospital; NH = Ngwelezana Hospital; ER/PR = estrogen receptor/progesterone receptor.

### Survival Analysis

**Figure 2.**
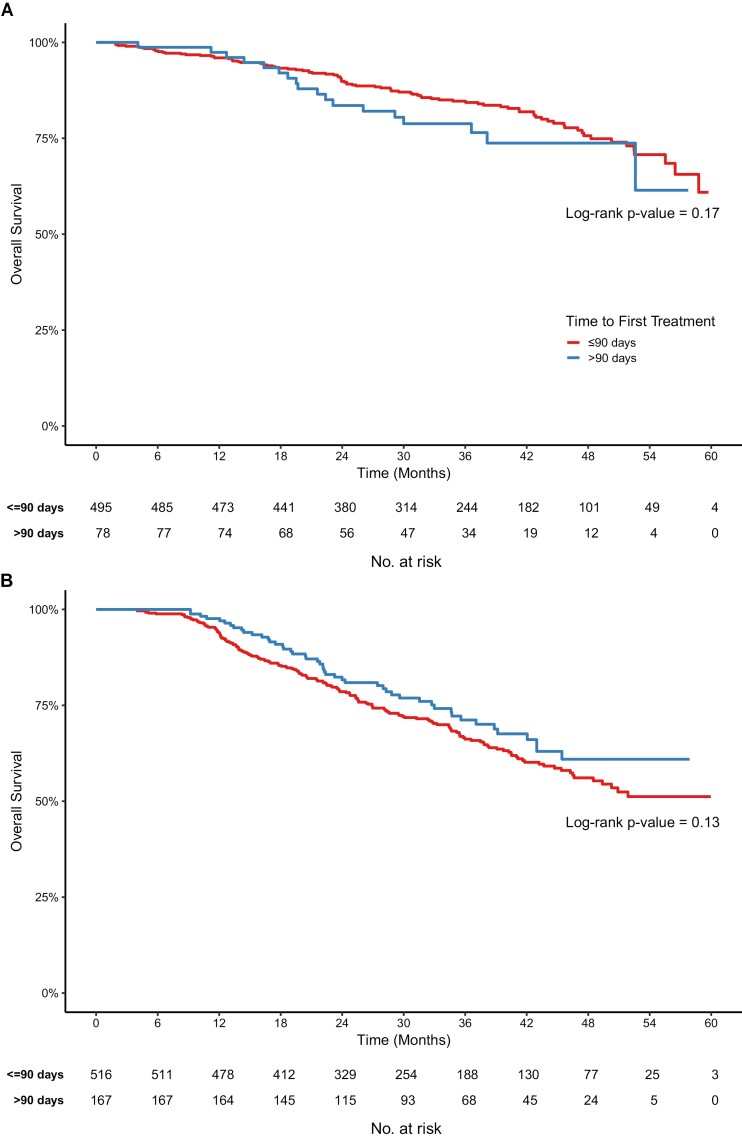
Overall survival in women in the SABCHO cohort with stages I-III breast cancer and treated with (**A**) upfront surgery and (**B**) neoadjuvant chemotherapy, by time to treatment initiation.

In the upfront surgery group, the 3-year OS was 85.0% and 78.8% for TTI ≤90 and >90 days, respectively (*P = .*17); in the NAC group, the 3-year OS was 66.3% and 71.6% for TTI ≤90 and >90 days, respectively (*P = .*13; Fig. 2). For women who had upfront BC surgery, multivariate analysis adjusted for patient, health system, and clinical factors showed that every 30-day delay in TTI was associated with an 11% increase in the risk of death (HR 1.11 [95%CI 1.003-1.22]; Table 3). The same multivariate analysis for women in the NAC group showed no effect of TTI on survival (HR for death 0.99 [95%CI [0.89-1.10] for every 30-day delay in TTI; Table 3).

**Table 3.** Cox proportional hazards ratio model for overall survival of women in the SABCHO cohort treated for stages I-III breast cancer, stratified by first treatment modality.

Characteristics	Primary surgery *N* = 543	Neoadjuvant chemotherapy *N* = 602
	HR [95%CI]	HR [95%CI]
Time to treatment initiation (by 30-day increments)	**1.11 [1.003-1.22]**	0.99 [0.89-1.10]
Age (by year)	1.00 [0.98-1.02]	1.01 [1.00-1.02]
Ethnicity		
Black	1.00 [ref]	1.00 [ref]
Other	1.07 [0.59-1.93]	0.99 [0.67-1.50]
Distance from hospital (by km)	1.00 [1.00-1.00]	1.00 [1.00-1.00]
Time to BC diagnosis		
<90 days	1.00 [ref]	1.00 [ref]
≥90 days	0.70 [0.44-1.12]	0.99 [0.74-1.32]
BC stage		
I-II	1.00 [ref]	1.00 [ref]
III	**2.91 [1.95-4.34]**	1.26 [0.83-1.93]
BC grade		
1-2	1.00 [ref]	1.00 [ref]
3	1.58 [0.98-2.37]	**2.00 [1.47-2.73]**
ER/PR status		
Positive	1.00 [ref]	1.00 [ref]
Negative	0.86 [0.50-1.49]	1.14 [0.83-1.56]
HER2 status		
Positive	0.64 [0.39-1.06]	1.28 [0.96-1.72]
Negative	1.00 [ref]	1.00 [ref]
Ki-67		
≤20%	1.00 [ref]	1.00 [ref]
>20%	1.28 [0.79-2.07]	0.94 [0.68-1.30]
HIV status		
Positive	**1.77 [1.08-2.90]**	**1.72 [1.22-2.42]**
Negative	1.00 [ref]	1.00 [ref]
Hospital		
CHBAH	1.00 [ref]	1.00 [ref]
CMJAH	0.89 [0.49-1.61]	0.83 [0.57-1.22]
GH	1.02 [0.60-1.74]	1.30 [0.80-2.11]
ALH	0.80 [0.32-1.96]	0.95 [0.59-1.53]
NH	—	1.00 [0.48-2.10]
Place of diagnosis		
Tertiary hospital	1.00 [ref]	1.00 [ref]
Other	0.77 [0.47-1.26]	1.12 [0.74-1.69]

Bold text indicateds statistically significant.

SABCHO = South Africa Breast Cancer and HIV Outcomes study; CHBAH = Chris Hani Baragwanath Academic Hospital; CMJAH = Charlotte Maxeke Johannesburg Academic Hospital; GH = Grey’s Hospital; ALH = Addington Hospital/Inkosi Albert Luthuli Central Hospital; NH = Ngwelezana Hospital; ER/PR = estrogen receptor/progesterone receptor.

**Figure 3.**
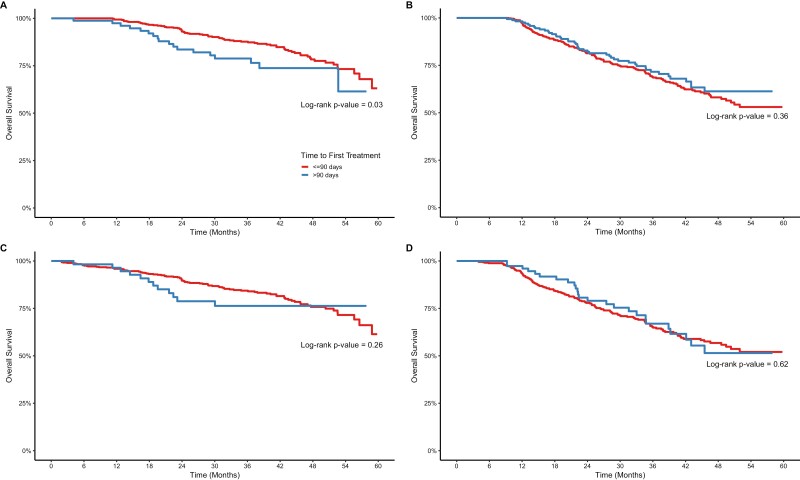
Overall survival in women in the SABCHO with stages I-III breast cancer and treated with (**A**) upfront surgery, conditional on surviving 1 year, (**B**) neoadjuvant chemotherapy, conditional on surviving 1 year, (**C**) upfront surgery and excluding women treated with ALH, and (**D**) neoadjuvant chemotherapy and excluding women treated at ALH.

Figure 3 depicts the -Kaplan-Meier curves for the 2 exploratory analyses: 1-year conditional survival analysis (exploratory 1) and survival analysis excluding women treated at ALH (exploratory 2). For the upfront surgery group, TTI ≤90 days was associated with improved survival compared with >90 days in the 1-year conditional survival analysis (Fig. 3A; *P = .*03). On multivariate analysis, the exploratory analyses again revealed that incremental 30-day delays in TTI were associated with worse survival in the upfront surgery group, although the result was not statistically significant for exploratory 2 (HR 1.15 [95%CI 1.05-1.27] and HR 1.10 [95%CI 0.99-1.22], for exploratories 1 and 2, respectively; Table 4). In the NAC group, the exploratory analyses found a trend toward worse survival with every 30-day delay in TTI, but this did not reach statistical significance (Table 4).

**Table 4.** Cox proportional hazards ratio model for overall survival of women in the SABCHO cohort treated for stages I-III breast cancer, stratified by first treatment modality and exploratory analysis.

Delay group	Primary surgery		Neoadjuvant chemotherapy	
	Exploratory 1^a^*N* = 524	Exploratory 2^a^*N* = 515	Exploratory 1^a^*N* = 585	Exploratory 2^a^*N* = 470
	HR [95%CI]	HR [95%CI]	HR [95%CI]	HR [95%CI]
Time to ­treatment initiation (by 30-day increments)	**1.15 [1.05-1.27]**	1.10 [0.99-1.22]	1.01 [0.92-1.12]	1.05 [0.93-1.18]

Bold text indicateds statistically significant.

Exploratory 1: conditional on surviving to 1 year.

Exploratory 2: Excluding patients from ALH Hospital.

a All models adjusted for: age, ethnicity, distance from hospital, time to BC diagnosis, BC stage, BC grade, Ki-67, HIV status, treatment hospital, and place of diagnosis.

## Discussion

Delays in treatment initiation were common among women treated for stage I-III BC at 6 public hospitals in SA, with a median TTI of 40 days (IQR 27-63) for women in the upfront surgery group and a median TTI of 63 days (IQR 45-90) for women in the NAC group. These findings are consistent with prior work by our group and others, finding significant delays along the continuum of BC care in SSA.^6,11-13,23,27^ Compared with studies from the US, women in the SABCHO cohort were almost twice as likely to experience treatment delays of >90 days (19.5% vs 10% or less in studies analyzing BC patients from the SEER-Medicare database).^16,28^ Regression analysis revealed that relative to CHBAH, patients at ALH, GH, and NH had longer treatment delays, as did those living further away from a treatment hospital and those who had ER/PR positive BC. A higher Ki-67 proliferation score and having upfront surgery were negatively correlated with treatment delays. Nonetheless, our multiple regression model explained only 19% of the variance in TTI in this cohort, suggesting that many unmeasured factors affect TTI in this population.

On multivariate survival analysis we found that among women treated with upfront surgery, every 30-day delay from BC diagnosis to surgery was associated with an 11% increase in the risk of death (HR 1.11 [95%CI 1.003-1.22]). In contrast, among women treated with NAC, delays in TTI were not associated with survival (Tables 3 and 4).

Our results are consistent with studies conducted in high-income medical settings, where delays from BC diagnosis to surgery are associated with worse survival, particularly in patients with stages I-II BC. Using NCDB and SEER-Medicare data from the US, Bleicher et al found that among >200 000 women with localized BC, each additional month of delay from diagnosis to surgery was associated with a 9-10% relative increase in all-cause mortality.^16^ On subgroup analysis, the effect of surgical delays on OS was limited to patients with stages I-II, but not stage III, BC. Polverini et al used a larger subset of NCDB data (>400 000 women) and also showed that surgical delays were associated with worse OS in women with stages I and II BC (19% and 16% respective increase in mortality with delays of more than 3 months), but not stage III BC.

While our analysis did not reveal an association between TTI and survival among women treated with NAC, a study by Gagliato et al of 5137 patients with localized BC treated at MD Anderson Cancer Center demonstrated worse survival among women who initiated NAC >60 days after BC diagnosis (HR 1.28 [95%CI 1.06-1.54] compared to ≤30 days).^19^ In stratified analysis, the association of NAC initiation >60 days after BC diagnosis and survival was only significant among women with stages I-II BC (HR 1.41 [95%CI 1.07-1.86], compared with ≤30 days), with no effect observed among women with stage III BC (HR 1.15 [95%CI 0.89-1.49]).^19^

One possible explanation why results from the NAC group enrolled in SABCHO are not consistent with published literature from the US is the difference in patient populations. The overall SABCHO patient population is markedly different from the BC patients included in US studies—women enrolled in SABCHO were relatively young (median age 52.3 years), majority Black (80%), mostly diagnosed with stage III BC (60%), and 23% were women living with HIV. These differences were most pronounced in the NAC group, where 81.5% of women were diagnosed with stage III BC. In contrast, in the study by Galgiato et al, only 36.2% of patients had stage III BC. We hypothesize that the lack of correlation between TTI and survival among women treated with NAC in the SABCHO cohort is because women with early-stage BC were under-represented in this group, and the negative impact of treatment delays is greatest in patients with early-stage BC, as suggested by Gagliato et alet al, Bleicher et al, and Polverini et al.

Meanwhile the upfront surgery group was enriched for older women with stages I-II, low-grade, ER/PR positive, HER2 negative BC, more closely approximating the patient populations included in the Bleicher et al and Polverini et al studies. As expected, women in SABCHO who underwent upfront surgery had improved survival relative to the higher-risk women treated with NAC (crude 3-year OS 78.8-85.0% for upfront surgery and 66.3-71.6% for NAC). Our finding that delays to BC surgery are associated with increased risk of death is generally consistent with prior literature, which suggests that delays to first BC treatment (surgery or NAC) are more likely to have an impact on the survival of lower-risk than higher-risk BC patients.

Another potential bias that may have affected our findings is understaging, whereby women with metastatic BC may have been misclassified as having localized BC because of less routine use of computerized tomography and magnetic resonance imaging. We address this possibility by performing a 1-year conditional survival analysis; this exploratory analysis increased the HR point-estimate for TTI in both the surgery and NAC group, suggesting the presence of some misclassification bias (Table 4, exploratory 1). Baseline characteristics of the women included in our study also showed that more than half of the women treated at ALH experienced treatment delays >90 days. Albert Luthuli Central Hospitals serves the KwaZulu Natal region of SA, which has a large rural population, more complex healthcare referral patterns than the other SABCHO sites, and experienced high medical provider attrition during part of the SABCHO enrollment period, all of which may account for the greater TTI observed at ALH.^29^ However, our exploratory survival analysis excluding the ALH site did not meaningfully change our main findings in either treatment group (Table 4, exploratory 2).

Lastly, our linear regression model explained only 19% of the variance in TTI in our cohort, and we cannot exclude the possibility that unmeasured patient behaviors and health system factors that led to longer TTI are also unmeasured confounders in our survival analysis. For example, in our survival analysis, we control for time from breast symptom awareness to BC diagnosis, level of education, and distance from treatment hospital, but these variables alone are unlikely to fully capture differences in patient behaviors and circumstances that can impact survival. Other unmeasured variables that may have impacted survival include poverty, access to care, and family support, among others. We were also unable to adjust our survival analysis for key health system and clinical factors, including chemotherapy dose intensity, chemotherapy completion, or surgery quality, all critical treatment factors known to impact survival. These are limitation of the current study, and we are actively collecting prospective data on chemotherapy dose intensity and completion among women with localized BC to address this knowledge gap.

Despite these limitations, our analysis uses one of the largest prospective databases of BC cases in a SSA country, with very little missing data, and excellent survival follow up, both of which are unique in this setting. Patients were also recruited from multiple public hospitals in SA, making our results more generalizable than single institution studies.

## Conclusion

Our study reveals that almost twice as many women with non-metastatic BC in the SABCHO cohort experienced treatment delays of >90 days, compared with women in the US (19.5% vs <10%). For women who had upfront BC surgery, TTI (in 30-day increments) was associated with an increased risk of death (HR 1.11 [95%CI 1.003-1.22]), while delays in TTI were not significantly associated with survival in higher-risk BC patients treated with NAC. Our findings demonstrate that reducing treatment delays complements the BC down-staging efforts promoted by the Global Breast Cancer Initiative, as improving such delays is most likely to improve BC survival among patients with earlier stage BC.

## Data Availability

The datasets generated during and/or analyzed during the current study are available from the corresponding author on reasonable request.
